# Short Versus Regular Periods of Cast Immobilization for Distal Radial Fractures: A Systematic Review and Meta-Analysis

**DOI:** 10.7759/cureus.54704

**Published:** 2024-02-22

**Authors:** Iqbal F Sayudo, Jesica P Sudarman, André Fernandes, Jae Yong Park, Liron Leibovitch, Elcio Machinski, Mohamed O Mahmoud

**Affiliations:** 1 Medicine, Syiah Kuala University, Banda Aceh, IDN; 2 Medicine, Atma Jaya Catholic University of Indonesia, Jakarta, IDN; 3 Trauma and Orthopaedics, York and Scarborough National Health Service (NHS) Trust, York, GBR; 4 Medicine and Surgery, Imperial College London, London, GBR; 5 Medicine, Bar-Ilan University, Safed, ISR; 6 Orthopaedics, State University of Ponta Grossa, Ponta Grossa, BRA

**Keywords:** conservative treatment, cast immobilization, wrist fractures, radius fractures, distal radial fractures

## Abstract

The current research on the recommended durations for cast immobilization in adults with distal radial fractures (DRFs) lacks a clear consensus or definitive conclusion. The standard practice involves casting immobilization for five to six weeks. The debate revolves around the potential benefits of shorter periods (three to four weeks) without compromising patient outcomes. While previous research has delved into this subject through systematic reviews, our study stands out by conducting a meta-analysis, aiming for a more precise understanding of whether short or regular cast immobilization duration proves more effective for treating DRFs.

A systematic search was conducted across PubMed, Embase, and the Cochrane Library databases to identify relevant studies. The focus was on comparing the outcomes of DRFs between short (three to four weeks) and regular (five to six weeks) periods of cast immobilization. The evaluated parameters include the shortened disabilities of the arm, shoulder, and hand questionnaire (quick (q) DASH); patient-rated wrist evaluation (PRWE); visual analog scale (VAS) score after cast removal; total complications; and the occurrence of complex regional pain syndrome (CRPS). Data synthesis employed the random-effects models, presenting the results as mean difference (MD) and weighted odds ratio (OR) with corresponding 95% confidence intervals (CI).

We included three randomized controlled trials (RCTs) with 252 patients, of whom 125 (49.6%) were immobilized in a cast for three to four weeks. The average age of participants was 61.20 years, and the follow-up duration was one year. The QDASH scores were significantly lower at 12 weeks (MD -6.72; 95% CI -10.76 to -2.69; p = 0.001), six months (MD -4.46; 95% CI -7.42 to -1.50; p = 0.003), and one year (MD -4.89; 95% CI -7.45 to -2.33; p = 0.0002) in patients treated with short periods compared to those with regular periods. The PRWE scores at six months (MD -2.33; 95% CI -8.10 to 3.43; p=0.43) did not significantly differ between groups. Also, the PRWE scores were significantly lower at one year (MD -4.93; 95% CI -9.03 to -0.82; p = 0.02) in the shorter cast-immobilization-period group. There were no significant differences in VAS score after cast removal, total complications, or CRPS.

The meta-analysis of RCTs on DRFs reveals that shorter periods of cast immobilization lead to better patient-reported functional outcomes (qDASH and PRWE). This suggests a potential benefit of reducing the immobilization duration for DRF patients, offering clinicians valuable insights for improved patient care and informed decision-making in clinical practice.

## Introduction and background

Current practice typically involves casting immobilization for periods ranging from five to six weeks, a conventional approach that has become a standard in the field. This established practice aims to promote fracture healing, reduce pain, and enhance overall functional outcomes for individuals with distal radial fractures (DRFs) [1]. However, amidst this prevailing practice, questions arise regarding the optimal duration of cast immobilization. The controversy centers around whether shorter periods of immobilization could offer comparable benefits without compromising patient outcomes [2].

While previous research has explored this topic through systematic reviews, our study distinguishes itself as a meta-analysis. This entails a more in-depth analysis of the data, going beyond mere summarization. Instead of simply condensing existing studies, we aim to integrate their findings to achieve a more precise understanding of whether short or regular periods of cast immobilization work better for DRFs [3].

Our primary aim is to address the ongoing discourse surrounding the optimal duration of cast immobilization for DRFs in a precise and informed manner. To accomplish this, our methodology involves a thorough examination of the existing body of literature. We intend to discern whether the effectiveness of a short casting period, ranging from three to four weeks, stands on par with the conventional practice of five to six weeks of immobilization. This meticulous analysis seeks to provide nuanced insights, offering guidance to healthcare professionals and patients alike. By basing our conclusions on the most recent and robust evidence, we aspire to contribute substantively to the understanding of the most effective treatment approach for DRFs.

## Review

Methods

Search Strategy

This systematic review and meta-analysis were conducted according to the guidelines outlined in the Cochrane Collaboration Handbook for Systematic Review of Intervention and the Preferred Reporting Items for Systematic Reviews and Meta-Analysis (PRISMA) statement [4,5]. We systematically searched PubMed, Embase, and the Cochrane library from inception to December 20, 2023, using the following search terms: ‘wrist fractures’, ‘distal radius fractures’, ‘cast’, ‘immobilization’, ‘conservative treatment’, ‘non-operative’, ‘nonoperative’, ‘nonsurgical’, ‘randomized’, ‘randomized controlled trial’, ‘controlled clinical trial’, and ‘placebo’.

Eligibility Criteria and Study Selection

This meta-analysis specifically included studies that met the eligibility criteria: (1) randomized trials; (2) comparing three to four weeks versus five to six weeks of cast immobilization; (3) enrollment of patients with DRFs; (4) a follow-up of at least one year; and (5) reports on any outcome of interest. The following are the exclusion criteria: (1) studies that lacked a control group; (2) participants <18 years old; (3) participants who underwent surgical treatment for DRFs; (4) conference abstracts, editorials, letters, review articles, case reports, non-English studies, and animal studies. References from all included studies, as well as those from previous systematic reviews, were manually examined to identify any additional relevant studies. Two authors (I.F.S. and J.P.S.) independently collected data based on predetermined search criteria and conducted quality assessments. The prospective meta-analysis protocol was registered on the International Prospective Register of Systematic Reviews (PROSPERO) under protocol #CRD42023495162 on December 30, 2023.

Data Extraction

The authors collected the following information from the studies: (1) baseline characteristics for the included studies; (2) primary outcomes, including patient-rated wrist evaluation (PRWE) and the shortened disabilities of the arm, shoulder, and hand questionnaire (quick (q) DASH) scores at 12 weeks, six months, and one year; and (3) secondary outcomes, including visual analog scale (VAS) scores after cast removal, total complications, and the rate of complex regional pain syndrome (CRPS).

Quality Assessment

The assessment of the risk of bias was independently conducted by two authors (I.F.S. and A.F.). Both authors utilized the risk of bias tool for randomized trials (ROB-2) as recommended by Cochrane [6]. In instances where discrepancies arose, a consensus was reached through discussion, addressing the reasons behind any disagreements.

Data Analysis

Categorical endpoint treatment effects were evaluated using odds ratios (OR) and 95% confidence intervals (CI), whereas mean differences (MD) were applied for continuous outcome comparisons. The DerSimonian and Laird random-effects models were used for the analysis. The missing mean and standard deviation (SD) were calculated from the median and interquartile range (IQR), according to Luo et al. [7]. Statistical analyses were conducted using Review Manager 5.4 (The Cochrane Collaboration, London, UK).

Assessment of Heterogeneity

Heterogeneity was evaluated through the Cochran Q test and I2 statistics. The significance of heterogeneity was established with p-values below 0.10 and I2 exceeding 25%. The interpretation of the results followed the recommendations outlined in the Cochrane Handbook for Systematic Reviews of Interventions [4].

Results

Literature Search

**Figure 1:**
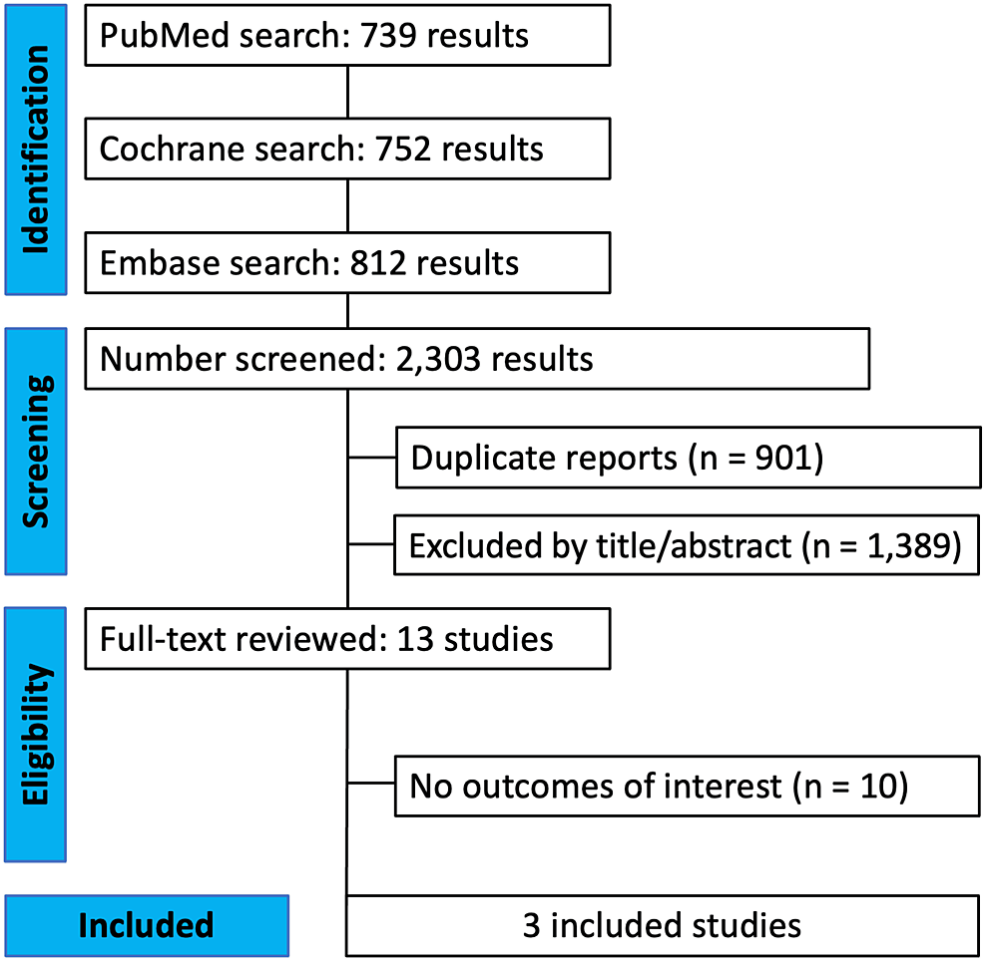
The PRISMA flow diagram for systematic review PRISMA: Preferred Reporting Items for Systematic Reviews and Meta-Analysis

As illustrated in Figure 1, the initial search yielded 2,303 results. Following the removal of duplicate records and studies that did not satisfy the inclusion criteria, 13 records remained for comprehensive review. From this selection, a total of three studies involving 252 patients were ultimately included. Among them, 125 (49.6%) patients were treated with short periods, and 127 (50.4%) were treated with regular periods of cast immobilization.

Characteristics of the Included Studies and Quality Assessment

Baseline characteristics are reported in Table 1. The analysis included three studies comparing three to four weeks with five to six weeks of cast immobilization. The mean age was 61.20 years, with a follow-up duration of one year.

**Table 1:** Baseline characteristics of included studies Categorical variables are presented as numbers and percentages, i.e., n (%). Continuous variables are presented as means ± SDs *SD values are estimated from group IQRs RCT: Randomized controlled trial; DRFs: Distal radial fractures; ND: Non- or minimally-displaced; DR: Displaced and reduced; NR: Not reported

Characteristics	Bentohami et al. [2] (n = 72)		van Delft et al. [8] (n = 100)		Elbardesy et al. [9] (n = 80)	
DRF type	ND		DR		ND	
Total follow-up in years	1		1		1	
Arms	3 weeks (n= 36)	5 weeks (n=36)	4 weeks (n=49)	6 weeks (n=51)	4 weeks (n=40)	6 weeks (n=40)
Female sex	26 (72)	23 (63)	40 (82)	45 (88)	32 (80)	32 (80)
Age (in years)	52.60 (30.44) *	59.44 (13.41) *	66 (59.25) *	69.10 (46.66) *	58.90 (NR)	64.76 (NR)
Dominant hand fractured	NR	NR	22 (51)	26 (52)	35 (87)	30 (75)

The assessment of the risk of bias was conducted using the ROB-2. Each study [2,8,9] received a score of low, moderate, high, or unclear risk of bias in five domains, namely selection, performance, detection, attrition, and reporting biases. Details of the risk of bias are presented in Table 2.

**Table 2:** Risk of bias for included studies

Study	Bias from randomization process	Bias due to deviations from intended interventions	Bias due to missing outcome data	Bias in measurement of the outcomes	Bias in selection of the reported result	Overall risk of bias
Bentohami et al. [2]	Low	Low	Low	Low	Low	Low
van Delft et al. [8]	Some concerns	Low	Low	Low	Low	Moderate risk
Elbardesy et al. [9]	Low	Low	Low	Low	Low	Low

Results From the Study

**Figure 2:**
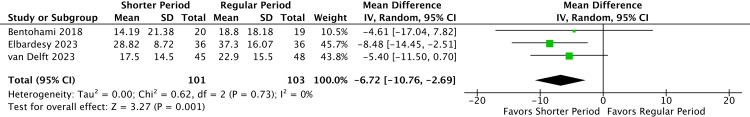
The qDASH scores at 12 weeks qDASH: Quick disability of arm, shoulder, and hand; IV: Information value Bentohami et al. [2]; van Delft et al. [8]; Elbardesy et al. [9]

**Figure 3:**
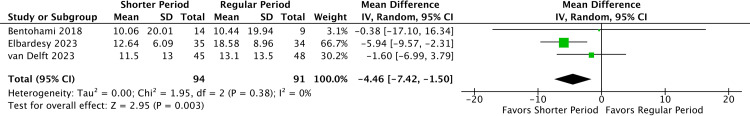
The qDASH scores at six months qDASH: Quick disability of arm, shoulder, and hand; IV: Information value Bentohami et al. [2]; van Delft et al. [8]; Elbardesy et al. [9]

**Figure 4:**
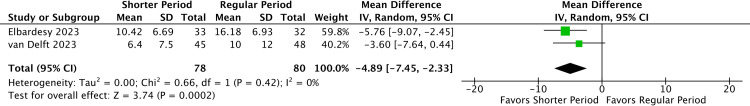
The qDASH scores at one year qDASH: Quick disability of arm, shoulder, and hand; IV: Information value van Delft et al. [8]; Elbardesy et al. [9]

Patients who underwent short periods of cast immobilization demonstrated significantly lower qDASH scores at 12 weeks (MD -6.72; 95% CI -10.76 to -2.69; p = 0.001) (Figure 2) compared to those with regular immobilization periods. Additionally, at six months (MD -4.46; 95% CI -7.42 to -1.50; p = 0.003) (Figure 3) and one year (MD -4.89; 95% CI -7.45 to -2.33; p = 0.0002) (Figure 4), the qDASH scores were significantly lower in the group with short cast immobilization periods.

**Figure 5:**
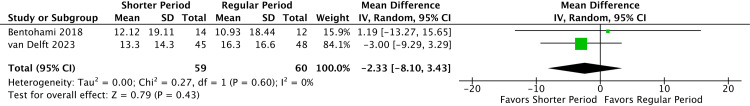
The PRWE scores at six months PRWE: Patient-rated wrist evaluation; IV: Information value Bentohami et al. [2]; van Delft et al. [8]

**Figure 6:**
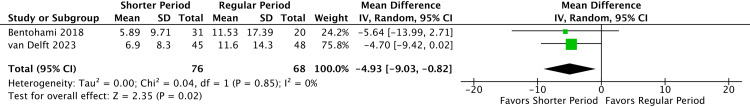
The PRWE scores at one year PRWE: Patient-rated wrist evaluation; IV: Information value Bentohami et al. [2]; van Delft et al. [8]

The PRWE scores showed no significant distinctions at six months between the two groups (MD -2.33; 95% CI -8.10 to 3.43; p = 0.43) (Figure 5). In the group with short periods of cast immobilization, PRWE scores showed a significant decrease at one year (MD -4.93; 95% CI -9.03 to -0.82; p = 0.02) (Figure 6).

**Figure 7:**
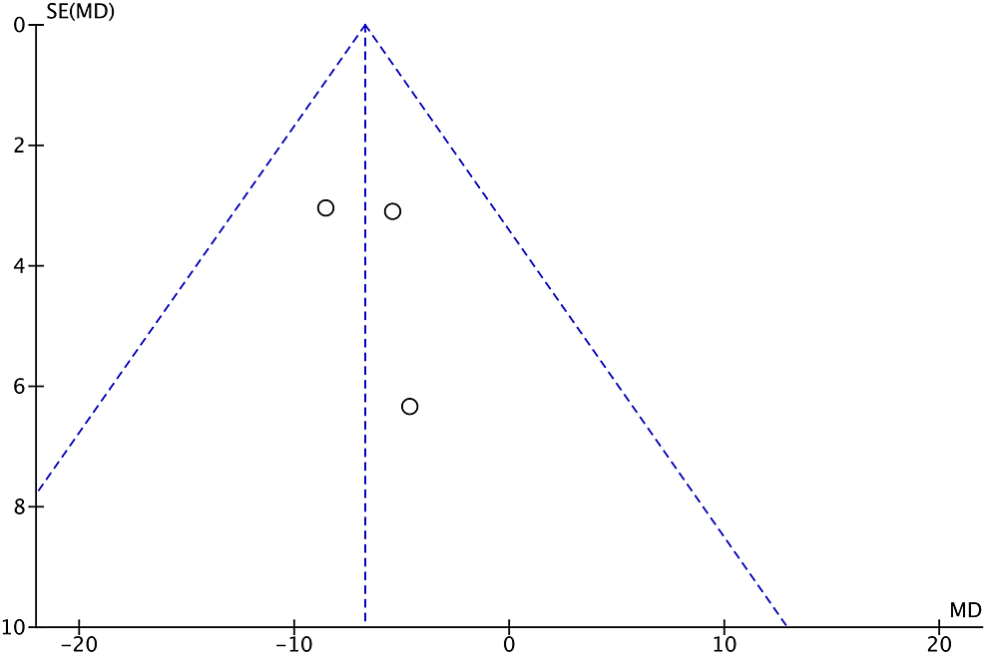
Funnel plot of qDASH scores at 12 weeks qDASH: Quick disability of arm, shoulder, and hand

**Figure 8:**
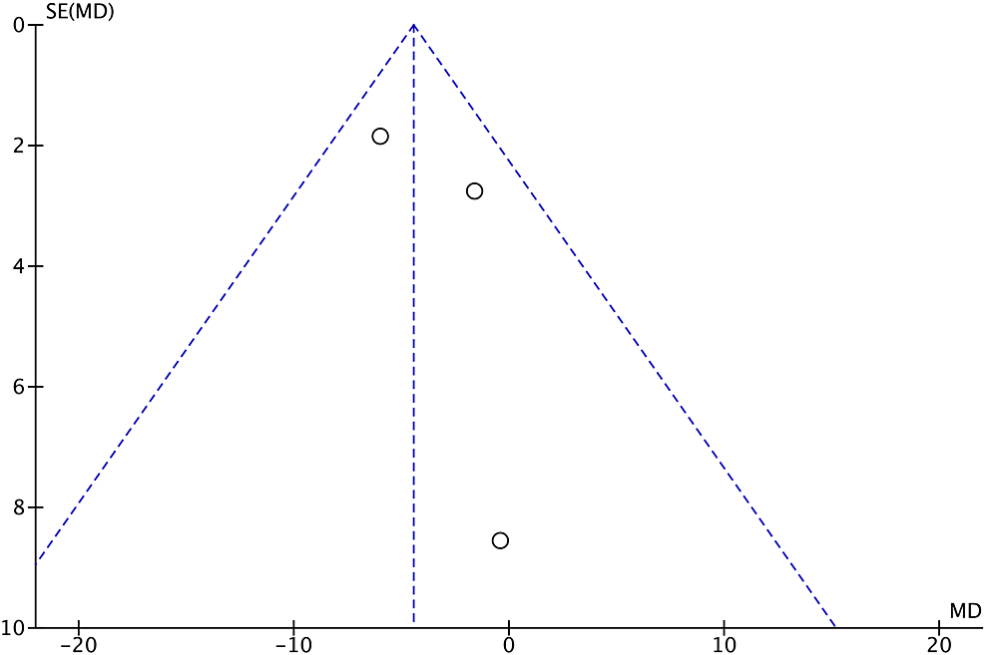
Funnel plot of qDASH scores at six months qDASH: Quick disability of arm, shoulder, and hand

**Figure 9:**
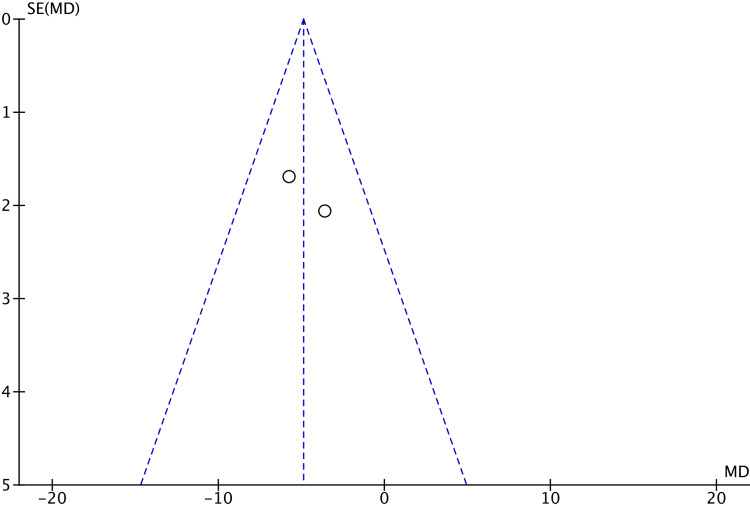
Funnel plot of qDASH scores at one year qDASH: Quick disability of arm, shoulder, and hand

**Figure 10:**
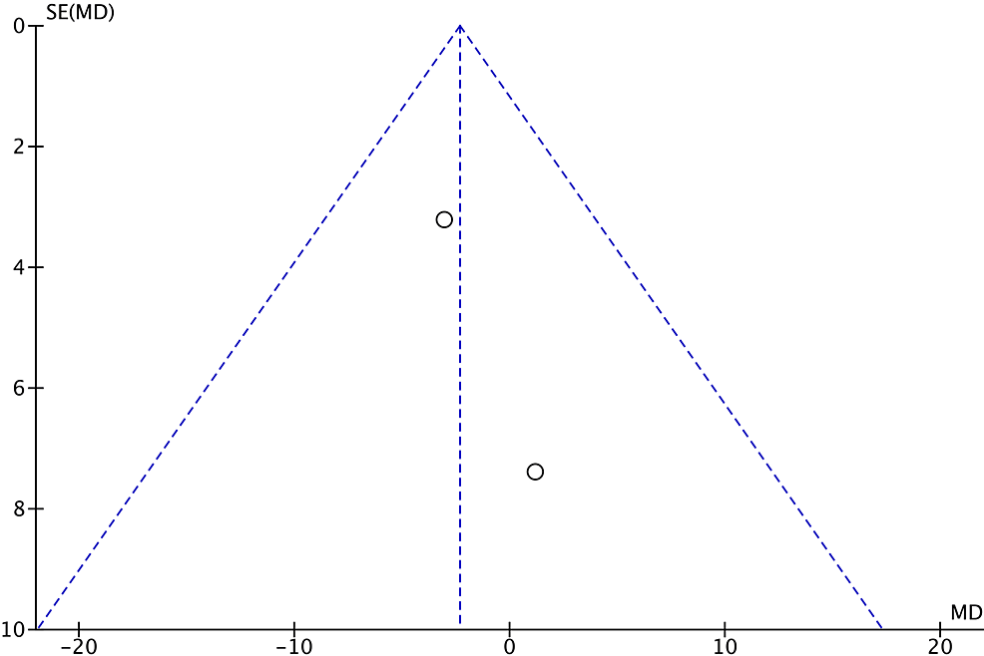
Funnel plot of PRWE scores at six months PRWE: Patient-rated wrist evaluation

**Figure 11:**
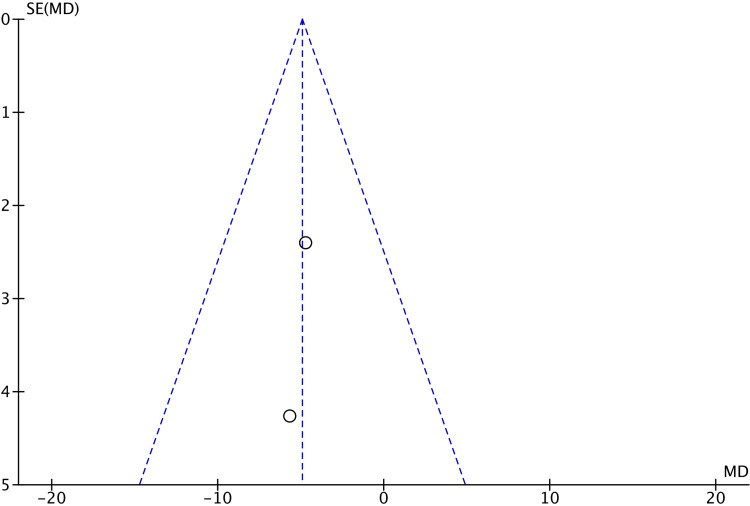
Funnel plot of PRWE scores at one year PRWE: Patient-rated wrist evaluation

There were no significant differences in VAS scores after cast removal (MD 0.66; 95% CI -1.00 to 2.31; p=0.44), total complications (OR 0.61; 95% CI 0.18 to 2.10; p=0.43), or the presence of CRPS (OR 0.43; 95% CI 0.06 to 3.04; p=0.40) between the two groups. The assessment of publication bias through funnel plot analysis (Figures 7-11) is restricted to scenarios with a small number of trials. Nevertheless, there was no definitive indication of publication bias found in this case.

Discussion

In this systematic review and meta-analysis, we included a total of three randomized clinical trials involving 252 patients. These studies analyzed homogenous data and documented patient-reported outcomes using qDASH and PRWE scores, VAS scores, total complications, and the presence of CRPS. In this study, we compared short periods ranging from three to four weeks with regular periods ranging from five to six weeks of cast immobilization. The main findings include: (1) a significantly lower qDASH score at 12 weeks, six months, and one year for patients treated with short periods of cast immobilization; (2) no significant difference between the groups in PRWE scores at six months, with a significantly lower PRWE score at one year in the group treated with short cast immobilization periods; (3) VAS score after cast removal, total complications, and presence of CRPS were not significantly different between the short and regular period groups.

Handoll et al. published a Cochrane review in 2003 comparing various conservative interventions for treating DRFs in adult patients. The review reported various outcomes, including pain, patient assessment, grip strength, complications, and functional grading. Despite the review being revised in 2008, the conclusion remained unchanged, as no further updates were implemented [1].

A previous systematic review concluded that reducing the duration of cast immobilization to three weeks or less did not result in inferior patient-reported functional outcomes, VAS scores, or radiological outcomes compared to the standard immobilization period [3]. Several recent studies have contributed to the understanding of patient-reported functional outcomes in DRFs. Boersma et al. compared the effects of a one-week to four- to five-week plaster cast immobilization, showing a decrease in scores for DASH and PRWE, yet this outcome did not display statistical significance between the two groups [10]. In a randomized controlled trial (RCT) conducted by Olech et al. on elderly patients, a lower mean Mayo wrist Sscore was reported in the four-week group compared to the six-week group; however, this difference was not statistically significant [11].

Christersson et al. conducted a recent RCT comparing 10 days to the regular period of cast immobilization in DRFs using the Gartland and Werley score. The groups showed similar scores, but there were no statistically significant differences [12]. A study by Christensen et al. in 1995 investigated patients with displaced and reduced DRFs, examining the impact of both three-week and five-week immobilization periods through the use of the Gartland and Werley score. Nine months post-immobilization, both groups exhibited excellent patient-reported outcomes, but this outcome did not reach statistical significance [13]. Similarly, in 1987, McAuliffe et al. reported excellent patient-reported outcomes based on the Gartland and Werley score, which also did not attain statistical significance [14]. A study by Jensen et al. in 1997 analyzed and compared one-week and three-week periods of immobilization using the Gartland and Werley score, and there was no statistical difference between the groups [15]. However, it is important to note that these studies [13-15] using the Gartland and Werley score were undertaken before the year 2000, and further studies need to be conducted to evaluate this issue.

McAuliffe et al.’s study showed a mean VAS score after cast removal in favor of the short-period group with a statistically significant result [14]. Olech et al.’s study revealed a lower VAS score in the four-week group compared to the six-week group, but these differences did not reach statistical significance [11]. However, a study by Christersson et al. reported more pain in the short-term group, though this difference was not significant [12]. Boersma et al. reported a similar number of complications, including secondary displacement and CRPS; however, there were no statistically significant differences [10]. 

Our study has limitations. The findings were influenced by the relatively small sample size in each study, the restricted availability of research, and the inability to perform a quantitative analysis for the PRWE score at 12 weeks due to insufficient data. Therefore, further research is essential to provide more definitive conclusions regarding the optimal period of immobilization in DRFs. Future studies should prioritize the use of randomized trials with larger and more diverse populations to thoroughly examine the comparison between shorter and regular immobilization periods for DRFs.

## Conclusions

In this meta-analysis of RCTs comparing short with regular periods of cast immobilization in patients with DRFs, the former duation was associated with better patient-reported functional outcomes, as indicated by the qDASH and PRWE scores. Therefore, it is advisable to consider shortening the period of immobilization for patients with DRFs. In conclusion, these findings provide clinicians with valuable insights, enriching their understanding of the optimal duration for immobilization in DRFs. This enhanced knowledge contributes to improved patient care and decision-making in clinical practice.
